# Retrospective Analysis of Risk Factors and Prediction Model of Cervical Anastomotic and Intrathoracic Anastomotic Leakage After Radical Esophagectomy

**DOI:** 10.1002/cnr2.70286

**Published:** 2025-09-16

**Authors:** Yao Wang, Jianning Xu, Jun Qian, Jian Qiu, Min Zhu, Daquan Wang

**Affiliations:** ^1^ Department of Cardiothoracic Surgery, Yancheng First Hospital Affiliated Hospital of Nanjing University Medical School Yancheng China

**Keywords:** anastomotic leak, esophageal cancer, postoperative complications, prediction model

## Abstract

**Background:**

Esophagogastric anastomotic leakage is a common complication after esophageal cancer surgery. Therefore, how to treat and predict is the focus of clinicians' research.

**Aims:**

To analyze the risk factors of cervical and intrathoracic anastomotic leakage after radical resection of esophageal cancer and establish a prediction model to provide a basis for early clinical prevention and treatment.

**Methods:**

We retrospectively analyzed the clinical data of 776 esophageal squamous cell carcinoma patients who underwent Sweet (*n*=115), Ivor‐Lewis (*n*=278), and McKeown (left neck anastomosis) (*n*=383) esophagectomy at Yancheng First People's Hospital from August 2019 to December 2021. Univariate and logistic regression models were used to analyze the independent risk factors of anastomotic leakage after esophageal cancer surgery, and a nomogram prediction model was established.

**Results:**

Of the 776 patients, 95 experienced postoperative anastomotic leakage, with an incidence of 12.2%. There were 63 cases of cervical anastomotic leakage and 32 cases of intrathoracic anastomotic leakage. The multivariate logistic regression analysis showed that BMI, high blood pressure, chronic bronchitis, peptic ulcer, operation way, anastomotic location, and postoperative albumin were independent risk factors for postoperative anastomotic leakage of esophageal cancer (*p* < 0.05). Our nomogram prediction model yielded a high predictive value, with an area under the receiver operating characteristic curve of 0.765 (95% CI 0.716–0.814).

**Conclusion:**

The occurrence of anastomotic leakage after esophageal cancer surgery is related to various factors, including BMI, hypertension, chronic bronchitis, peptic ulcer, operation way, anastomotic way, postoperative albumin, and anastomotic location. The clinical prediction model can promote the early detection, diagnosis, intervention, and treatment of anastomotic leakage and shorten the hospitalization time of patients.

## 1 Introduction

Esophageal cancer is one of the most common malignant tumors in China; surgical treatment remains the mainstay of treatment [1, 2, 3]. At present, the surgical approach mainly includes left thoraco‐esophageal carcinoma radical resection (Sweet), right thoraco‐abdominal two‐incision radical resection of esophageal carcinoma (Ivor‐Lewis) and total endoscopic radical resection of esophageal carcinoma (McKeown, left cervical anastomosis). However, irrespective of the surgical method adopted, the incidence of anastomotic leakage remains relatively high. Esophagogastric anastomotic leakage (EGAL) is the most common and serious complication after esophageal cancer surgery, with an average incidence of about 0.8%–6.5% in China and a mortality rate as high as 11.0%–35.7% [4], which represents a significant threat to the postoperative safety of patients. In the present study, 95 cases of anastomotic leakage were observed in 776 esophageal squamous cell carcinoma patients that underwent radical resection. The present study retrospectively analyzed the clinical data of patients who underwent esophageal cancer surgery in our hospital from August 2019 to December 2021; we summarized the independent risk factors for postoperative EGAL and established a prediction model to provide a theoretical basis for the clinical prevention and treatment of EGAL.

## 2 Materials and Methods

### 2.1 Clinical Information

The clinical data of 776 patients with esophageal squamous cell carcinoma that underwent radical resection in Yancheng First People's Hospital from August 2019 to December 2021 were retrospectively analyzed. Among them, 115 patients (100 males and 15 females) with a mean age of 68.83 ± 8.39 years were treated with Sweet esophagectomy, and postoperative leakage occurred in 13 cases. 278 patients (216 males and 62 females), with a mean age of 68.75 ± 7.08 years, underwent Ivor‐Lewis esophagectomy, and anastomotic leakage occurred in 19 cases [5]. Finally, 383 cases (324 males and 59 females), with a mean age of 69.3 ± 7.39 years, underwent McKeown esophagectomy, and anastomotic leakage was observed in 63 cases. The inclusion criteria were: (1) Esophageal squamous cell carcinoma confirmed by preoperative gastroscopic biopsy; (2) No tumor invasion of adjacent vital organs, and no metastasis to distant lymph nodes; (3) Patients that could tolerate surgery. The exclusion criteria included: (1) Patients with other malignant tumors; (2) Patients with a poor cardiac or pulmonary function that could not tolerate surgery; (3) Patients with coagulation disorders.

### 2.2 Data Collection

The clinical data of patients were retrospectively analyzed. The patient's gender, age, past medical history, body mass index (BMI), preoperative albumin, American Society of Anesthesiologists (ASA), operation time, operation method, tumor location, tumor size, tumor stage, anastomosis location, postoperative albumin, chylothorax, and pulmonary infection were recorded in our hospital's electronic medical record information system. In terms of the staging and segmentation of esophageal cancer, it can refer to the TNM staging standard (8th edition) of esophageal cancer in the International Union Against Cancer (UICC) in 2017. The patient records/information were anonymized and deidentified prior to analysis, and informed consent was not obtained from the participants due to the retrospective nature of the study by the Clinical Research Ethics Committee of the Yancheng No. 1 People's Hospital (K‐045).

### 2.3 Surgical Approach

Sweet esophagectomy: After induction of general anesthesia, the right decubitus position was taken, and an incision was made through the left fifth intercostal space to access the thoracic cavity. Then, the esophagus was dissociated, lymph nodes were dissected, the diaphragm was opened, the stomach was dissociated, lymph nodes in the abdominal cavity were dissected, and a tubular stomach was made. The tubular stomach was pulled above or below the aortic arch for anastomosis. The gastric tube and jejunal nutrition tube were placed. The thoracic cavity was closed, and one thoracic and one mediastinal drainage tube were inserted.

Ivor‐Lewis esophagectomy: After induction of general anesthesia, the patient was placed in the supine position, and an incision was made from the xiphoid process to the umbilicus to access the abdominal cavity. A tubular stomach was created, abdominal lymph nodes were dissected, and an abdominal drainage tube was placed before abdominal closure. The patient was then placed in the left decubitus position, and an incision was made in the right 5th intercostal space to enter the right thoracic cavity. The relationship between the size and location of the mass and surrounding tissues was determined. The esophagus was then dissociated, mediastinal lymph nodes were dissected, and the tubular stomach was pulled to the azygous vein arch or under the arch for anastomosis. The gastric tube and jejunal nutrition tube were placed. The thoracic cavity was closed, and one thoracic and one mediastinal drainage tube were inserted.

McKeown esophagectomy: After the patient was placed in a left decubitus position and induction of general anesthesia, four small incisions were made in the right chest. An incision was made in the 7th intercostal space in the middle axillary line for the camera port. Further incisions were made in the 4th intercostal space on the axillary line, the 8th intercostal space in the posterior axillary line, and the 6th intercostal space in the axillary line for the working ports. The mediastinal pleura was opened, the thoracic esophagus was fully dissociated, mediastinal lymph nodes were dissected, and the stomach was removed in the supine position by laparoscopy. Abdominal lymph nodes were dissected, and a small incision was made under the xiphoid process to create a tubular stomach. Finally, an oblique incision was made in the left neck, the cervical esophagus was dissociated, and the tubular stomach was pulled to the neck for anastomosis. The gastric tube and jejunal nutrition tube were placed. The thoracic cavity and abdominal cavity were closed. A neck incision was made, and a drainage tube was inserted in the thoracic cavity, mediastinum, neck, and abdomen.

### 2.4 Clinical Diagnosis of Anastomotic Leakage

The occurrence of EGAL after esophageal cancer surgery was used as an outcome indicator, and the patients were divided into an anastomotic leakage (AL) group and a non‐anastomotic leakage (no‐AL) group. Anastomotic leakage is divided into early, intermediate, and late stages [6, 7], occurring 1–3 days, 4–14 days, and more than 2 weeks after surgery, respectively. Fifty‐one cases of anastomotic leakage showed symptoms such as elevated body temperature, leukocytosis, and chest tightness. Intrathoracic anastomotic leakage is characterized by chest and mediastinal drainage tubes draining yellow muddy viscous liquid or food residue. On chest CT examination, an encapsulated effusion or pneumothorax may be observed. After oral administration of methylene blue, a blue liquid was observed in the drainage tube, or contrast agent leakage was observed during upper gastrointestinal tract angiography, and the leakage location was determined. Cervical anastomotic leakage is characterized by incision redness and swelling, fluctuating sensation, purulent fluid oozing after suture removal, accompanied by sputum, saliva, and food residue effusion. Oral methylene blue and upper gastrointestinal angiography can be used to assist in the diagnosis.

### 2.5 Clinical Treatment

For patients with cervical anastomotic leakage, the neck suture was first removed, the incision was opened to ensure smooth drainage, and kept clean and dry. Antibiotic treatment was provided, and the patient was kept nil per os with gastrointestinal decompression. Adequate enteral nutrition and intravenous infusion were given to maintain water and electrolyte homeostasis. For patients with thoracic anastomotic leakage, the chest and mediastinal drainage tubes were kept patent and flushed with normal saline or metronidazole when necessary. If there was an encapsulated pleural effusion, puncture drainage was performed under ultrasound guidance. Simultaneously, under the guidance of intervention, the internal drainage tube was placed, and the external drainage tube was continuously sucked with low negative pressure. Chest CT was reexamined about 1 week; if the internal drainage tube did not drain effusion, the internal drainage tube was withdrawn 2 cm every other day until the internal drainage tube was completely withdrawn. In addition, antibiotic treatment was provided, and the patient was in abrosia with gastrointestinal decompression. Adequate enteral nutrition and intravenous infusion were given to maintain water and electrolyte homeostasis.

### 2.6 Statistical Analysis

R 4.1.2 software was used to establish the database and conduct data analysis. Qualitative variables were described by frequency and rate, and comparisons between groups were performed by the Chi‐square test or Fisher's exact test. Normally distributed continuous variables were represented by mean and standard deviation, and the independent sample *t*‐test was used to compare the two groups. Continuous variables that did not conform to normal distribution were represented by median (lower and upper quartile), and comparisons between the two groups were performed by Mann Whitney *U* test. The independent risk factors of anastomotic leakage were analyzed by multivariate logistic regression, and a linear prediction model was established. The Area Under Receiver Operating Characteristic (ROC) Curve (AUC) was used to evaluate the model's performance. The calibration of the model was evaluated by the Homer Lemeshoe test, and a calibration curve was drawn. A *p*‐value < 0.05 was statistically significant.

## 3 Results

### 3.1 Anastomotic Leakage After Esophagectomy

AL was observed in 12.2% (95/776) of patients that underwent esophagectomy, including cases of intermediate leakage (*n* = 87) and late leakage (*n* = 8). Among them, there were four patients who died of massive hemorrhage of the digestive tract, two patients who died of multiple organ failure, eight patients who were discharged because their families gave up treatment, and the remaining 81 patients who were discharged after conservative treatment.

The diagnosis of AL after esophageal cancer is based on the clinical symptoms and examination; although the diagnosis can be challenging, especially in the early stage of concealed leakage, where the leak is small, and the clinical symptoms are not obvious, resulting in very difficult diagnosis [8]. Huang et al. [9] reported that multiple oral contrast agents are needed to establish a definitive diagnosis of hidden leakage. In addition, one patient experienced contralateral thoracic leakage at the anastomotic site, exhibiting persistent fever. The chest CT examination suggested a small amount of contralateral thoracic effusion, and no leakage was found after ingesting oral contrast. Purulent malodorous fluid was aspirated from the contralateral thoracic cavity under B‐ultrasound guidance to establish the diagnosis. Therefore, leakage should be suspected in cases of fever of unknown cause and contralateral pleural effusion.

Esophagotracheal leakage occurred in 1 patient with intrathoracic anastomotic leakage, which could be diagnosed by bronchoscopy and gastrointestinal angiography. The patient was transferred to Zhongshan Hospital in Shanghai and recovered. However, the application of coated stents in such cases is subject to much controversy. Indeed, the release of the stent may exacerbate the leakage and affect healing. On the other hand, the infection may aggravate due to poor drainage. Hoeppner et al. [10] reported that stent‐associated complications were as high as 71%. Therefore, caution should be taken when using film‐covered stents.

### 3.2 Group Comparison

As shown in Table 1, the results of group comparison showed that BMI, high blood pressure, chronic bronchitis, smoking, diabetes, drinking, peptic ulcer, NACT, coagulation, operation way, anesthesia, operation time, the lymph node cleaning number, anastomosis way, anastomotic location, tumor location, and postoperative albumin were 17factors with statistical significance between the two groups (*p* < 0.05).

**TABLE 1:** Group comparison.

Variables	No‐AL group	AL group	*χ* ^2^/*Z*	*p*
Gender			2.124a	0.145
Male	545 (80.0%)	82 (86.3%)		
Female	136 (20.0%)	13 (13.7%)		
BMI			12.256a	0.002
18–24	466 (68.4%)	48 (50.5%)		
< 18	48 (7.0%)	12 (12.6%)		
> 24	167 (24.5%)	35 (36.8%)		
High blood pressure	136/681 (20%)	36/95 (37.9%)	15.525a	< 0.001
Coronary disease	63/681 (9.3%)	10/95 (10.5%)	0.159a	0.690
Arrhythmias	29/681 (4.3%)	7/95 (7.4%)	/b	0.190
Chronic bronchitis	95/681 (14.0%)	28/95 (29.5%)	15.063a	< 0.001
Smoking	360/681 (52.9%)	61/95 (64.2%)	4.325a	0.038
Diabetes	170/681 (25.0%)	33/95 (34.7%)	4.123a	0.042
Drinking	199/681 (29.2%)	38/95 (40.0%)	4.566a	0.033
Peptic ulcer	39/681 (5.7%)	13/95 (13.7%)	8.444a	0.004
Thoracic and abdominal surgery	53/681 (7.8%)	11/95 (11.6%)	1.588a	0.208
NACT	35/681 (5.1%)	10/95 (10.5%)	4.429a	0.035
Coagulation			6.299a	0.012
Normal	595 (87.4%)	74 (77.9%)		
Abnormal	86 (12.6%)	21 (22.1%)		
Preoperative total protein level	64.29 (60.67, 67.45)	64.12 (59.91, 66.69)	0.974c	0.330
Preoperative albumin	41.19 (38.38, 43.70)	41.06 (38.42, 43.80)	0.132c	0.895
ASA grade			1.500a	0.472
Level 1	67 (9.8%)	11 (11.6%)		
Level 2	246 (36.1%)	39 (41.1%)		
Level 3	368 (54.0%)	45 (47.4%)		
Operation way			13.970a	0.001
Ivor‐Lewis	259 (38.0%)	19 (20.0%)		
Sweet	102 (15.0%)	13 (13.7%)		
McKeown	320 (47.0%)	63 (66.3%)		
Anesthesia			4.558a	0.033
Single lumen tube	380 (55.8%)	64 (67.4%)		
Double lumen tube	301 (44.2%)	31 (32.6%)		
Operation time	223.00 (179.00, 276.00)	241.00 (199.00, 292.00)	−2.127c	0.033
The lymph node cleaning number	10.00 (7.00, 14.00)	11.00 (9.00, 15.00)	−2.038c	0.042
Anastomosis way			/b	0.004
Manual suture	19 (2.8%)	9 (9.5%)		
Mechanical suture	662 (97.2%)	86 (90.5%)		
Anastomotic location			5.330a	0.021
Cervical	366 (53.7%)	63 (66.3%)		
Intrathoracic	315 (46.3%)	32 (33.7%)		
Tumor location			6.061a	0.048
Upper section	74 (10.9%)	13 (13.7%)		
Middle section	291 (42.7%)	28 (29.5%)		
Lower section	316 (46.4%)	54 (56.8%)		
Anastomosis embedding	412/681 (60.5%)	49/95 (51.6%)	2.751a	0.097
Tumor stage			3.267a	0.195
I stage	74 (10.9%)	8 (8.4%)		
II stage	329 (48.3%)	39 (41.1%)		
III stage	278 (40.8%)	48 (50.5%)		
Postoperative albumin			8.112a	0.004
< 35 g	478 (70.2%)	80 (84.2%)		
≥ 35 g	203 (29.8%)	15 (15.8%)		
Tumor size			0.406a	0.524
< 3.5 cm	349 (51.2%)	52 (54.7%)		
≥ 3.5 cm	332 (48.8%)	43 (45.3%)		

*Note:* a, chi‐square test; b, Mann Whitney *U* test; c, Fisher's exact test.

### 3.3 Univariate Analysis and Multivariate Analysis of Risk Factors

As shown in Table 2, univariate analysis of AL after esophagectomy showed that BMI, high blood pressure, chronic bronchitis, smoking, diabetes, drinking, peptic ulcer, NACT, coagulation, operation way, anesthesia, the lymph node cleaning number, anastomosis way, anastomotic location, postoperative anemia, postoperative albumin, pulmonary infection, and chylothorax were potential risk factors for anastomotic leakage (*p* < 0.05).

**TABLE 2:** Univariate analysis and multivariate analysis of risk factors.

Variables	Univariable analysis		Multivariable analysis	
	Crude OR (95% CI)	*p*	Adjusted OR (95% CI)	*p*
Gender				
Male	Reference			
Female	0.635 (0.344–1.175)	0.148		
BMI				
18–24	Reference		Reference	
< 18	2.427 (1.207–4.882)	0.013	2.575 (1.198–5.536)	0.015
> 24	2.035 (1.272–3.256)	0.003	2.051 (1.244–3.382)	0.005
High blood pressure	2.445 (1.551–3.854)	< 0.001	2.369 (1.452–3.865)	0.001
Coronary disease	1.154 (0.570–2.335)	0.690		
Arrhythmias	1.788 (0.761–4.205)	0.183		
Chronic bronchitis	2.578 (1.577–4.214)	< 0.001	2.365 (1.390–4.024)	0.002
Smoking	1.600 (1.025–2.498)	0.039		
Diabetes	1.600 (1.013–2.526)	0.044		
Drinking	1.615 (1.037–2.513)	0.034		
Peptic ulcer	2.610 (1.338–5.092)	0.005	2.561 (1.242–5.280)	0.011
Thoracic and abdominal surgery	1.552 (0.780–3.088)	0.211		
NACT	2.171 (1.038–4.543)	0.040		
Coagulation				
Normal	Reference			
Abnormal	1.963 (1.150–3.352)	0.013		
Preoperative total protein level	0.977 (0.934–1.022)	0.319		
Preoperative albumin	0.998 (0.943–1.056)	0.948		
ASA grade				
Level 1	Reference			
Level 2	0.966 (0.469–1.987)	0.924		
Level 3	0.745 (0.367–1.513)	0.415		
Operation way				
Ivor‐Lewis	Reference		Reference	
Sweet	1.737 (0.827–3.648)	0.144	1.878 (0.862–4.090)	0.113
McKeown	2.684 (1.566–4.598)	< 0.001	2.756 (1.555–4.886)	0.001
Anesthesia				
Single lumen tube	Reference			
Double lumen tube	0.612 (0.388–0.964)	0.034		
Operation time	1.003 (1.000–1.006)	0.068		
The lymph node cleaning number	1.048 (1.001–1.098)	0.046		
Anastomosis way				
Manual suture	Reference		Reference	
Mechanical suture	0.274 (0.120–0.625)	0.002	0.182 (0.074–0.446)	< 0.001

Variables	Univariable analysis		Multivariable analysis	
	Crude OR (95% CI)	*p*	Adjusted OR (95% CI)	*p*
Anastomotic location				
Cervical	Reference		Reference	
Intrathoracic	0.590 (0.376–0.927)	0.022	0.672 (0.415–1.089)	0.106
Tumor location				
Upper section	Reference			
Middle section	0.548 (0.270–1.109)	0.094		
Lower section	0.973 (0.505–1.875)	0.934		
Anastomosis embedding	0.695 (0.452–1.070)	0.098		
Tumor stage				
I stage	Reference			
II stage	1.097 (0.492–2.444)	0.822		
III stage	1.597 (0.724–3.523)	0.246		
Postoperative albumin				
< 35 g	Reference		Reference	
≥ 35 g	0.442 (0.248–0.785)	0.005	0.449 (0.246–0.819)	0.009
Tumor size				
< 3.5 cm	Reference			
≥ 3.5 cm	0.869 (0.565–1.338)	0.524		

### 3.4 Multivariate Logistic Regression Analysis of Risk Factors

As shown in Table 3, indicators with statistical significance during univariate analysis were incorporated for multivariate logistic regression analysis. The results showed that BMI, high blood pressure, chronic bronchitis, peptic ulcer, operation way, anastomosis way, and postoperative albumin were independent risk factors for AL after esophageal cancer (*p* < 0.05).

**TABLE 3:** Multivariate logistic regression analysis of risk factors.

Variables	B	SE	Wald *χ* ^2^	OR (95% CI)	*p*
BMI					
18–24				Reference	
< 18	0.883	0.394	5.019	2.419 (1.117–5.240)	0.025
> 24	0.623	0.264	5.559	1.864 (1.111–3.128)	0.018
High blood pressure	0.790	0.257	9.443	2.203 (1.331–3.646)	0.002
Chronic bronchitis	0.841	0.277	9.185	2.319 (1.346–3.994)	0.002
Smoking	0.323	0.252	1.638	1.381 (0.842–2.263)	0.201
Diabetes	0.459	0.257	3.193	1.583 (0.956–2.621)	0.074
Drinking	0.329	0.250	1.736	1.390 (0.852–2.267)	0.188
Peptic ulcer	0.765	0.387	3.897	2.148 (1.005–4.589)	0.048
NACT	0.504	0.436	1.331	1.655 (0.703–3.893)	0.249
Coagulation					
Normal				Reference	
Abnormal	0.556	0.309	3.234	1.743 (0.951–3.195)	0.072
Operation way					
Ivor‐Lewis				Reference	
Sweet	0.525	0.409	1.651	1.691 (0.759–3.767)	0.199
McKeown	0.968	0.299	10.490	2.634 (1.466–4.733)	0.001
Anesthesia					
Single lumen tube				Reference	
Double lumen tube	−0.316	0.251	1.589	0.729 (0.446–1.192)	0.207
The lymph node cleaning number	0.040	0.025	2.547	1.041 (0.991–1.094)	0.110
Anastomosis way					
Manual Suture				Reference	
Mechanical Suture	−1.489	0.480	9.617	0.226 (0.088–0.578)	0.002
Anastomotic location					
Cervical				Reference	
Intrathoracic	−0.386	0.253	2.326	0.680 (0.414–1.116)	0.127
Postoperative albumin					
< 35 g				Reference	
≥ 35 g	−0.760	0.313	5.884	0.468 (0.253–0.864)	0.015
Constant	−6.524	1.086	36.095		< 0.001

### 3.5 Establishment of a Nomogram Prediction Model

**FIGURE 1:**
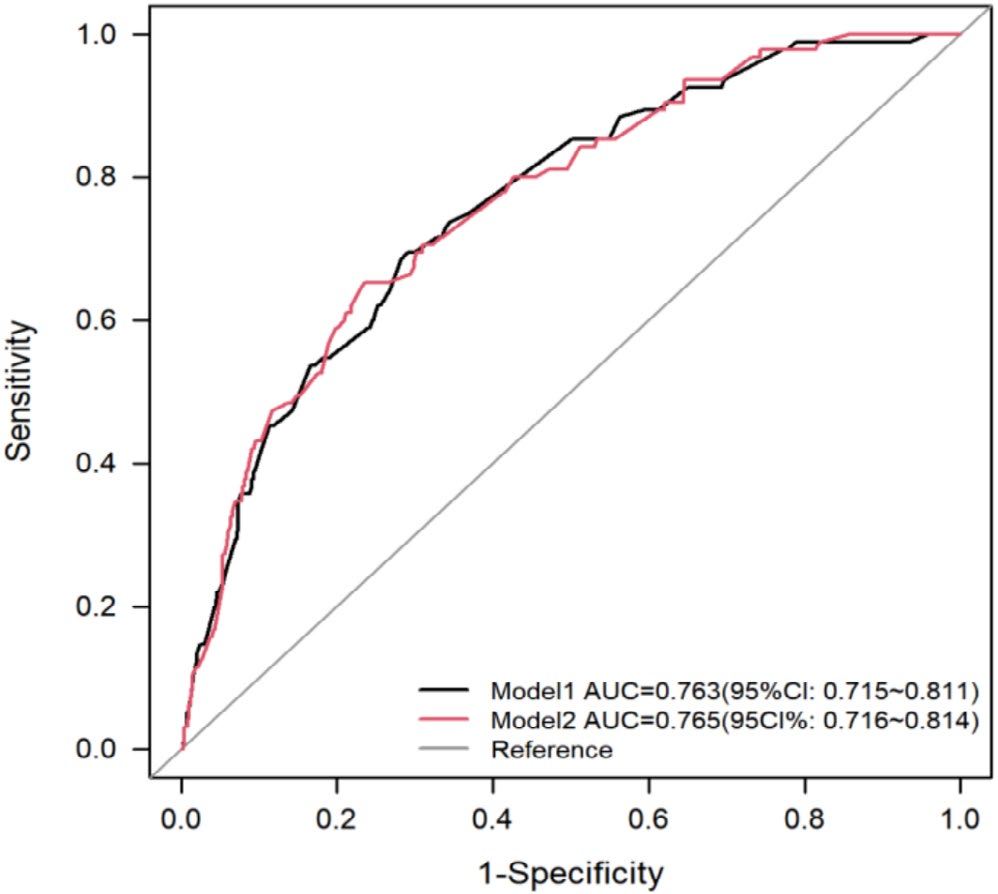
ROC curve.

**FIGURE 2:**
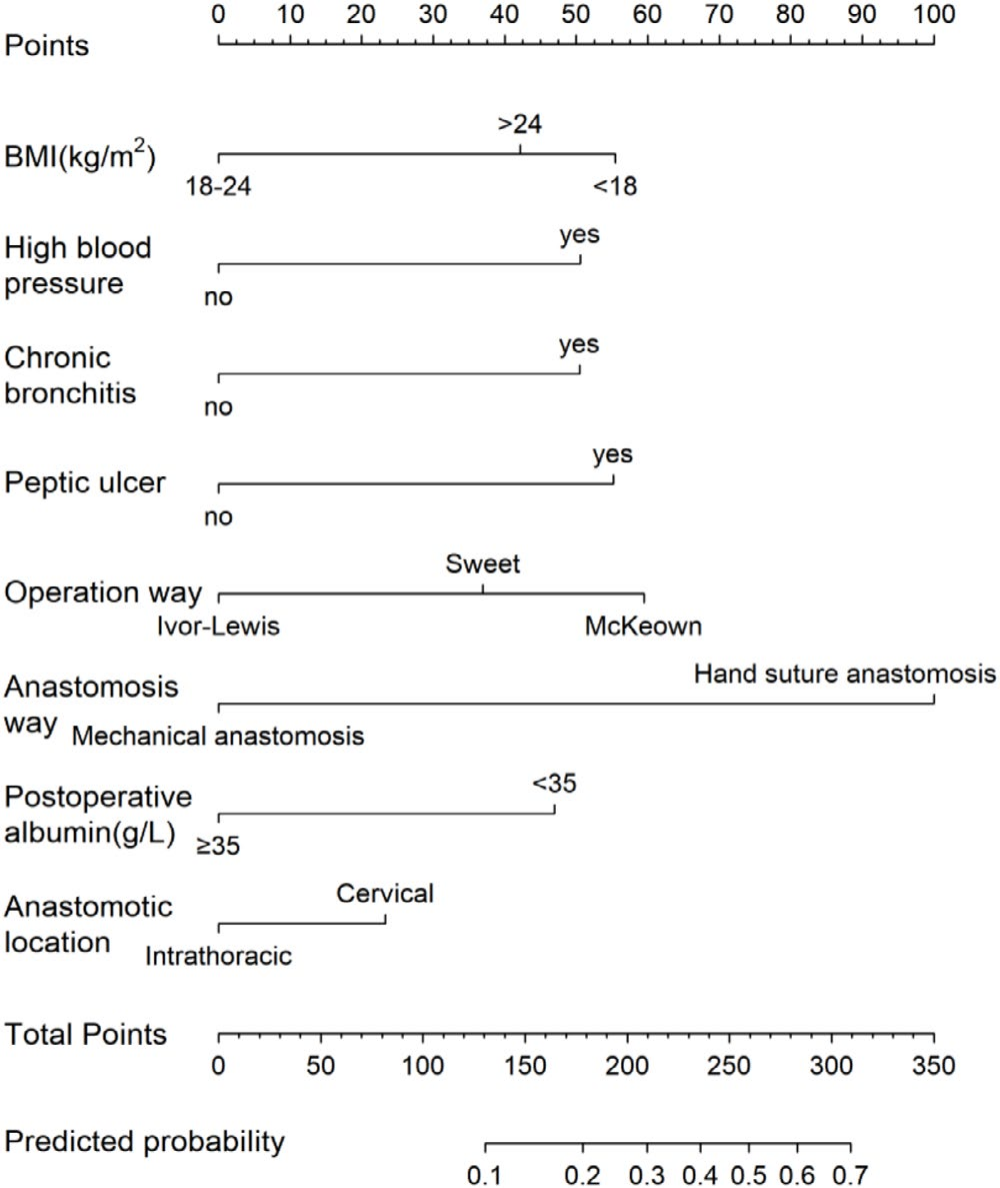
Nomogram prediction model.

**FIGURE 3:**
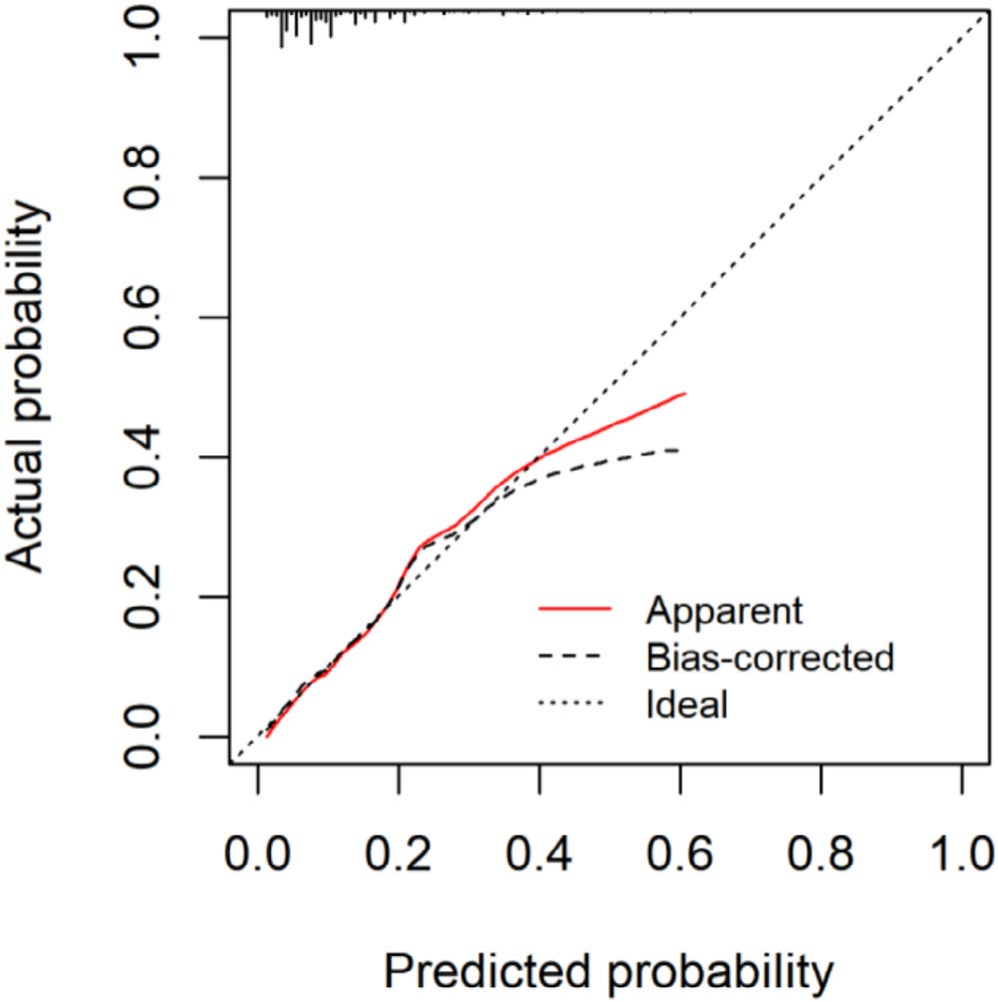
Calibration curve.

We established Model 1 based on seven independent risk factors obtained through multivariate logistic regression analysis. In addition, the anastomotic location was closely related to the anastomotic leakage in clinical practice. Therefore, we considered the clinical significance of anastomotic location and added Model 1 to establish Model 2. As shown in Figure 1, the area under the receiver operating characteristic curve (AUC) of Model 2 was 0.765 (95% CI 0.716–0.814), which was slightly higher than Model 1. As shown in Table 4, the accuracy of Model 2 is higher than that of Model 1. Although the sensitivity of Model 2 has decreased, the Youden index (sensitivity+specificity‐1) has increased due to its increased specificity. In summary, we have chosen Model 2 to establish a nomogram model (Figure 2). Our model achieved an AUC value of 0.765 (95% CI 0.719–0.815) after 1000 bootstrap resampling internal validations. Meanwhile, according to the calibration chart, the predicted probability did not significantly deviate from the actual probability. Moreover, on the basis of the Hosmer–Lemeshow test *χ* ^2^ = 6.145, *p* = 0.631, *p* value > 0.05, indicating that the model has good calibration (Figure 3).

**TABLE 4:** Diagnostic capabilities of model 1 and model 2.

Model	Accuracy	Sensitivity	Specificity	Youden	AUC (95% CI)
Model1	70.9%	0.695	0.711	0.405	0.763 (0.715–0.811)
Model2	75.1%	0.653	0.765	0.418	0.765 (0.716–0.814)

## 4 Discussion

### 4.1 Analysis of Risk Factors for EGAL

The occurrence of EGAL arises from multifactorial interactions during the perioperative period. Hypertension has been identified as a significant risk factor for AL in multivariate analysis. The pathophysiological mechanism may involve increased peripheral vascular resistance and microcirculatory impairment induced by hypertension, collectively reducing local blood perfusion at the anastomotic site. Vascular wall calcification in hypertensive patients further exacerbates peri‐anastomotic ischemia and hypoxia [11]. Therefore, strict blood pressure control and comprehensive preoperative evaluation are critical. The risk of EGAL is strongly correlated with a history of peptic ulcer disease [12]. These patients exhibit severe esophageal/gastric mucosal damage, reduced tissue elasticity, and weakened suture retention capacity. Concurrent *Helicobacter pylori* infection at ulcer sites elevates gastrin release, gastric acid secretion, and pepsinogen activity, collectively disrupting the anastomotic healing microenvironment [13]. Our data revealed significantly higher lymph node dissection counts in the AL group compared to the non‐AL group, suggesting that extensive lymphadenectomy (particularly three‐field dissection) may compromise anastomotic vascular perfusion [14], highlighting the need to balance oncological radicality with surgical safety. Chronic bronchitis has been confirmed as an independent EGAL risk factor [15, 16]. Postoperative pulmonary infections in these patients increase anastomotic tension through frequent coughing, while impaired pulmonary oxygenation and diffusion capacity predispose to tissue hypoxia and respiratory failure. Preoperative smoking cessation, pulmonary rehabilitation, and aggressive postoperative respiratory management constitute essential preventive measures.

With a lower mortality risk than intrathoracic leaks, it is essential to tailor surgical approaches to patient‐specific factors and surgeon expertise. Mechanical stapling was predominant in this study to minimize leakage risk, whereas manual layered anastomosis requires exceptional surgical skill. Technical imperfections during manual procedures, such as mucosal defects, ulceration, or improper suture tension (excessive tightness or looseness), may impair mucosal regeneration and anastomotic integrity. Optimal mucosal alignment remains critical for healing efficacy.

Most cases of anastomotic leakage (AL) can be managed conservatively through jejunal feeding, gastrointestinal decompression, nutritional support, adequate drainage, and infection control. This study identified postoperative serum albumin as an independent risk factor for AL following esophageal cancer surgery, consistent with findings by Xu Feng et al. [17] in minimally invasive procedures. Serum albumin serves as a critical marker of nutritional status. Preoperative malnutrition, anemia, and hypoproteinemia are common in esophageal cancer patients due to chronic dysphagia, while postoperative fasting exacerbates protein deficiency and metabolic imbalances, impairing anastomotic healing. Postoperative hypoalbuminemia directly impacts AL risk, underscoring the importance of early jejunal nutrition to restore protein levels. Exogenous protein supplementation enhances nutritional status, intestinal motility, and immune function while reducing infection rates and optimizing nutrient absorption, thereby effectively lowering AL incidence.

### 4.2 The Risk Prediction Model of EGAL

This study analyzed anastomotic leakage (AL) risk factors and developed a clinically applicable nomogram for risk assessment. Huang et al. [18] employed LASSO regression to identify seven predictors (sex, diabetes history, anastomosis type, reconstruction pathway, smoking history, arrhythmia, and postoperative C‐reactive protein), constructing a predictive model with internal validation that demonstrated strong performance. While LASSO regression addresses variable selection limitations and exhibits superior predictive accuracy compared to conventional methods, further statistical validation remains necessary. Using univariate and multivariate logistic regression, we identified eight predictors: BMI, hypertension, chronic bronchitis, peptic ulcer history, surgical approach, anastomotic technique, postoperative albumin levels, and anastomotic location. The model incorporates clinically accessible parameters, enabling AL probability evaluation by postoperative day 4, with an ROC‐AUC of 0.765, indicating robust predictive value.

In conclusion, anastomotic leakage (AL) remains the most frequent complication following radical resection of esophageal cancer. Advances in minimally invasive surgical techniques and enhanced recovery protocols have prioritized AL prevention and perioperative safety. Key strategies to reduce AL incidence include patient‐tailored surgical planning, operative time optimization, serum albumin maintenance, and rigorous respiratory management. The predictive model developed in this study provides clinicians with a tool for early risk assessment and targeted intervention, ultimately reducing AL occurrence.

This study has several limitations. Being a single‐center investigation with a limited sample size, it lacks external validation, potentially limiting the generalizability of the prediction model to other clinical settings. The prolonged data collection period and inclusion of multiple variables (some with residual confounding factors) may partially compromise the model's accuracy. Although these findings provide a foundational framework for AL risk prediction, further validation through multicenter studies with larger cohorts is necessary to confirm clinical applicability. Nevertheless, this model establishes a foundation for future research aimed at refining prediction tools to reduce postoperative AL incidence.

## Author Contributions

Y.W., J.X., and D.W. designed this study. M.Z. and J.Q. collected and collated the data. Y.W., J.Q., and J.X. analyzed and interpreted the data. Y.W. and D.W. completed the article. All authors read and approved the final manuscript.

## Ethics Statement

This retrospective study was approved by the Clinical Research Ethics Committee of the Yancheng No. 1 People's Hospital (K‐045).

## Conflicts of Interest

The authors declare no conflicts of interest.

## Data Availability

The corresponding author may provide the data used in this study upon reasonable request.
